# Digital Health and Big Data Analytics: Implications of Real-World Evidence for Clinicians and Policymakers

**DOI:** 10.3390/ijerph19148364

**Published:** 2022-07-08

**Authors:** Teresa Magalhães, Ricardo Jorge Dinis-Oliveira, Tiago Taveira-Gomes

**Affiliations:** 1 Department of Public Health and Forensic Sciences, and Medical Education, Faculty of Medicine, University of Porto, 4200-319 Porto, Portugal; 2 Center for Health Technology and Services Research (CINTESIS), 4200-450 Porto, Portugal; 3 MTG Research and Development Lab, 4200-604 Porto, Portugal; 4 TOXRUN—Toxicology Research Unit, University Institute of Health Sciences, Advanced Polytechnic and University Cooperative (CESPU), CRL, 4585-116 Gandra, Portugal; 5 UCIBIO-REQUIMTE, Laboratory of Toxicology, Department of Biological Sciences, Faculty of Pharmacy, University of Porto, 4050-313 Porto, Portugal; 6 Department of Community Medicine, Information and Decision in Health, Faculty of Medicine, University of Porto, 4200-319 Porto, Portugal; 7 Faculty of Health Sciences, University Fernando Pessoa (FCS-UFP), 4249-004 Porto, Portugal

**Keywords:** real-world evidence, real-world data, big data, digital health, healthcare

## Abstract

Real world data (RWD) and real-world evidence (RWE) plays an increasingly important role in clinical research since scientific knowledge is obtained during routine clinical large-scale practice and not experimentally as occurs in the highly controlled traditional clinical trials. Particularly, the electronic health records (EHRs) are a relevant source of data. Nevertheless, there are also significant challenges in the correct use and interpretation of EHRs data, such as bias, heterogeneity of the population, and missing or non-standardized data formats. Despite the RWD and RWE recognized difficulties, these are easily outweighed by the benefits of ensuring the efficacy, safety, and cost-effectiveness in complement to the gold standards of the randomized controlled trial (RCT), namely by providing a complete picture regarding factors and variables that can guide robust clinical decisions. Their relevance can be even further evident as healthcare units develop more accurate EHRs always in the respect for the privacy of patient data. This editorial is an overview of the RWD and RWE major aspects of the state of the art and supports the Special Issue on “Digital Health and Big Data Analytics: Implications of Real-World Evidence for Clinicians and Policymakers” aimed to explore all the potential and the utility of RWD and RWE in offering insights on diseases in a broad spectrum.

Real-world data (RWD) are currently collected from a variety of sources, namely electronic medical records (EMRs), claims and billing databases, product and disease registries, patient-generated data, and home medical devices for monitoring patients, such as the smartwatches. From RWD and through robust analytics, real-world evidence (RWE) can be produced with clear potential benefits for the health and outcomes of patients [1,2,3]. In other words, RWE offers a real difference between what is expected to happen and what is really happening specially in comparison to traditional clinical trials, whose well-known limitations of more homogeneous populations, make it difficult to generalize findings to larger scales. Examples of real-world health and medicine data include EMRs regarding patient demographics, family history, comorbidities, treatments, outcomes, and other information, the National Health Insurance claims data, cancer registry data, and reports on adverse drug reactions. Automated data abstraction (e.g., from EHRs) has also been shown to be highly accurate and faster than manual abstraction and its value may increase if more structured EHRs, high-quality datasets and terms accepted as being the gold standard definition are used; in other words this evolution can project RWE studies to an higher scale [4,5]. Indeed, the successful implementation of EHRs in institutions across the globe has opened a novel research era with new challenges for the scientific medical research community. Even in comparison to randomized controlled clinical trials (RCTs), RWE studies are emerging as a post-market surveillance approach to confer valuable complementing evidence specially aiming to identify rare adverse events or long-term effects of existing drugs due to their potential to analyze larger patient populations across longer timelines [6,7,8]. Recently our group has been exploring the usefulness of RWD and RWE in several fields of medicine, namely in studying the prevalence of heart failure, type 2 diabetes mellitus, chronic kidney disease, to name a few [9,10]. The Sentinel System [11] is an example of the extensive use of RWD to uncover adverse drug reactions. Recognizing the potential for additional use of RWD and RWE for regulatory and funding purposes, the United States Congress included in the 21st Century Cures legislation (Cures) (Public Law 114–225, 13 December 2016) the direction for Food and Drug Administration (FDA) to develop a program to evaluate the potential use of RWE to support the approval of a new indication of an approved drug or to support or satisfy post-approval study requirements. The potential benefits of leveraging RWD, stored in EHRs and other sources, have become even more evident with the COVID-19 pandemic either by evaluating the efficacy of vaccine, the risk of diabetes following COVID-19 or the course of COVID-19 in patients with lysosomal storage disorders [12,13,14].

In another emergent perspective, and as complement to the modern techniques available to forensic sciences to solve many cases, by incorporating big data analytics and RWE, a reshaping the world of forensics is expected, for example by developing predictive models of violence, such as domestic violence and accidents. Despite the huge amount of data generated in forensic cases, as recently highlighted, in the field of forensics, big data is still waiting for a comprehensive outline of its contribution to the field [15]. Additionally, insurance medicine is an importance source of RWD regarding diseases and accidents, not only in the traditional view of health but also in the social medicine perspective [1].

In the Special Issue entitled “Digital Health and Big Data Analytics: Implications of Real-World Evidence for Clinicians and Policymakers” (https://www.mdpi.com/journal/ijerph/special_issues/digital_big_data), we are interested in receiving original articles, reviews, technical notes, protocols, guidelines, etc., with no restriction on the length of the papers, exploring the usefulness of RWD and RWE in offering insights on diseases, regarding the pathophysiological aspects, new techniques for diagnosis, more safe treatments, novel preventive, and predictive models of diseases identification, evaluate the effectiveness of clinical guidelines, study the morbidity, mortality, and socioeconomic impact. However, much more can be generated from RWE to contribute to the medical knowledge from the regional up to the global level, ultimately leading to changes in healthcare policies and contribute to the sustainability of healthcare systems, especially if performed and respecting specific criteria of robustness and transparency to achieve high-quality evidence avoiding incorrect or unreliable conclusions. Despite RWE can virtually be generated from every healthcare institution around the globe, to reach the RWE expected outcomes, the setting in which evidence is generated and the methodologic approach used to conduct the surveillance or research are two critical dimensions to be considered.
